# 

**Published:** 1903-06-20

**Authors:** 


**Notices of Births, Deaths, and Marriages** are inserted in this column at a charge of 2s. 6d. for an announcement not exceeding four lines.

**DEATH.**

NURSE MARGARET J. N. HENDERSON, late of St. Thomas's Hospital, passed away on June 9th, 1903. Interment from Dunelm, Bridlington on Friday next at 2 o'clock. (2833)

### NOTICE TO CORRESPONDENTS.

All MSS., Letters, Books for Review, and other matters intended for the Editor, should be addressed THE EDITOR, "THE HOSPITAL," THE HOSPITAL BUILDINGS, 28 & 29 SOUTHAMPTON STREET, STRAND, W.C.

The Editor cannot undertake to return rejected MS., even when accompanied by stamped directed envelope.

**Notice to Advertisers and Subscribers.**

All Advertisements, Orders for Copies of THE HOSPITAL, and Business Communications should be addressed to **THE MANAGER, (Not to the Editor),** THE HOSPITAL, 28 & 29 Southampton Street, Strand, London, W.C.

The Cost of Subscription, post free, is as follows.—Per week, 3½d.; per quarter, 3s. 9d.; per half-year, 7s. 6d.; per annum, 15s. Foreign and Colonial:—Per annum, 19s. 6d., payable, in all cases, in advance.

**NOTICE.—Vol. XXXIII. of "The Hospital" (October 1902 to March 1903) in handsome cloth binding, will shortly be on sale at the office of this Journal, price 7s. 6d. Cases for binding the Numbers contained in this Volume 1s. 6d. Index to Vol. XXXIII., 2½d., post free.**

**The monthly part for June is now ready. Price 1s., by post 1s. 3d.**

### SCRAPS AND GLEANINGS.

A second death of a boy from nicotine poisoning caused by chewing tobacco has occurred at Tredegar.

By the will of the late Mr. John Wilder, of Reading, the Royal Berkshire Hospital receives a legacy of £500.

Since the passing of the new Licensing Act, there is stated to have been an increase of drunkenness in Manchester.

The London School Board have rejected a proposal in favour of compulsory school attendance up to 15 unless pupils pass the eighth standard before reaching that age.

The new isolation hospital buildings of the Ilfracombe Urban District Council have cost about £5,000. These new buildings comprise an isolation block, administrative block, and mortuary.

Two schemes are under consideration for improving the accommodation at the Stockton and Thornaby Hospital, especially with regard to the out-patients' department and the provision of a house for the medical officer. One of the schemes is that prepared by Mr. W. E. Henman, and is considered the better of the two; it will cost, however, £2,000 more than the other plan, making the total outlay on the proposed new accommodation between £20,000 and £25,000.

### THE STUDENT OF MEDICINE.

### APPOINTMENTS.

**C. H. Badcock**, B.A.Cantab., M.R.C.S., L.R.C.P., appointed House Surgeon to Salisbury Infirmary. **George H. Carrington**, M.R.C.S., L.S.A., appointed Medical Officer of Health for the Borough of Poole and to the Port Sanitary Authority. **C. T. Empey**, M.D., C.M.Kingston, L.R.C.P.Edin., appointed District Medical Officer of the Keighley Union. **F. Evered**, L.R.C.P., L.R.C.S.Edin., appointed District Medical Officer of the Westbourne Union. **R. J. Harris**, M.R.C.S., L.R.C.P.Lond., appointed District Medical Officer of the Rochdale Union. **G. H. Harvey**, M.B., C.M.Aberd., appointed District Medical Officer of the Parish of Camberwell. **J. R. Hutchinson**, M.B., Ch.B.Vict., appointed Junior Resident Medical Officer to the Chorlton Union Workhouse Infirmary, Withington. **Miss Helena G. Jones**, M.B., B.S.Lond., appointed Assistant Medical Officer of the Greenwich Union Infirmary. **Caroline Elizabeth O'Connor**, M.B., Ch.B.Edin., appointed Superintendent of Special Schools to the City of Birmingham Education Committee. **Beaumont Percival**, M.B.Cantab., appointed Physician to the Essex and Colchester General Hospital. **C. M. Phillips**, M.D.Brux., M.R.C.S., L.R.C.P., appointed District Medical Officer of the Parish of Bristol. **W. A. Rudd**, M.D.Durh., M.R.C.S.Eng., L.R.C.P.Lond., appointed Divisional Surgeon to the Metropolitan Police at Acton, **W. E. D. Telford**, M.A., B.C.Cantab., F.R.C.S.Eng., appointed Resident Surgical Officer to the Manchester Royal Infirmary. **E. Vallance**, M.R.C.S., L.R.C.P.Lond., appointed Medical Officer of the West Ham Union Infirmary. **A. E. White**, L.R.C.P., L.R.C.S.Edin., appointed District Medical Officer of the Barnsley Union.

### "The Hospital" Institutional Library.

"Hospitals and Asylums of the World." 4 Vols., with a Portfolio of Plans, complete £12 12s.

"Burdett's Hospitals and Charities, 1902." 5s.

"Burdett's Official Nursing Directory, 1903." 3s. net.

"Cottage Hospitals, General, Fever, and Convalescent." 10s. 6d.

"Uniform System of Accounts for Hospitals and Public Institutions." New Edition. 4s. net.

"Hospital Expenditure: The Commissariat." 2s. 6d.

"A Legal Handbook for the Use of Hospital Authorities." 2s. 6d.

"The Cottage Hospital Case Book or Register of Patients." 10s. and 12s. 6d.

"The Furnishing and Appliances of a Cottage." 6d.

All these are published by the Scientific Press, Ltd., and may be obtained through any bookseller, or direct from the publishers, 28 and 29 Southampton Street, Strand, London, W.C.

### For Hospital Matrons.

### HOSPITAL EXPENDITURE: The Commissariat.

Price **2/6** post free.

This book contains the most valuable information for the guidance of all who have to do with hospital housekeeping and the feeding of the resident staff, nurses, patients, and domestic household. With this assistance material economies can be introduced. The information given is collective and comparative, so that matrons can learn in the easiest manner the arrangements made in other institutions.

### ACCOUNT BOOKS AND REGISTERS FOR HOSPITALS AND INSTITUTIONS.

This series of books contains a complete set of account books and registers for large and small institutions. The Linen Register contains printed lists of hospital linen, ruled columns for stock, condemned, remaining, and issued linen, with details and space for remarks. This will be found invaluable. Full description on books and prices on application. Any book can be obtained separately.

### ART OF FEEDING THE INVALID.

By A MEDICAL PRACTITIONER & A LADY PROFESSOR of COOKERY.

Price **1/6** post free.

This little book contains a number of practical hints and receipts of great use to the hospital housekeeper.

### For Hospital Sisters.

### HOSPITAL SISTERS AND THEIR DUTIES.

By THE MATRON OF THE LONDON HOSPITAL.

Price **2/6** post free.

Miss Lückes' reputation in the nursing world is sufficient testimony that her book contains admirable information and advice to all about to take up a Sister's duties.

*These books are obtainable of all Booksellers, and are published by*
THE SCIENTIFIC PRESS, LIMITED, 28 & 29 Southampton Street, Strand, London,
*who will send their latest Catalogue of Nursing Text-books post free to any Nurse on application.*

